# Distance Learning Effects Among Italian Children and Parents During COVID-19 Related School Lockdown

**DOI:** 10.3389/fpsyt.2021.782353

**Published:** 2021-12-23

**Authors:** Giulia Crisci, Irene C. Mammarella, Ughetta M. M. Moscardino, Maja Roch, Lisa B. Thorell

**Affiliations:** ^1^Department of Developmental Psychology and Socialization, University of Padua, Padua, Italy; ^2^Division of Psychology, Department of Clinical Neuroscience, Karolinska Institutet, Stockholm, Sweden

**Keywords:** distance learning, COVID-19, executive functions, parents' well-being, children

## Abstract

**Background:** During the COVID-19 pandemic, both children and their parents experienced consequences related to distance learning (DL). However, positive and negative effects have varied greatly among families, and the specific factors explaining these differences in experiences are still underexplored. In this study, we examined children's executive functions (EF) and parents' psychological well-being in relation to negative and positive effects of DL on both children and their parents.

**Method:** Participants were 637 Italian parents (92% mothers) with a child (48% male) aged between 6 and 19 years involved in DL due to school closures during the first wave of the COVID-19 pandemic. Data were collected using an online survey. We performed three fixed-order hierarchical multiple regression analyses with child age and sex, children's EF deficits, and parents' psychological well-being as independent variables, and DL-related negative effects (on the child and on the parent) and DL-related positive effects as dependent variables.

**Results:** The results of the regression analyses showed that for negative effects of DL, younger age and greater EF deficits explained most part of the variance. Specifically, regarding negative effects on children, the most important factor was EF deficits, whereas regarding negative effects on parents, child age was the most important factor. For positive effects of DL, all variables explained only a small part of the variance. Child age was the most important factor, but EF deficits and parents' psychological well-being also had a significant impact.

**Conclusions:** The effects of DL during school closures vary widely across families. Our findings indicate that intervention efforts need to consider background variables, child factors, as well as parent factors when supporting families with homeschooling in times of pandemic.

## Introduction

### Effects of Distance Learning Among Italian Families During the COVID-19 Pandemic

Children, adolescents, and their parents have experienced important modifications to daily life activities due to the COVID-19 pandemic. Previous research has shown that school closures and the consequent distance learning (DL) have resulted in psychosocial problems for children (1, 2). In many countries, schools were not able to quickly adapt their teaching to an online format, which often caused increased levels of stress and worry for parents who suddenly had to take responsibility for the teaching role (3). However, there has been little research examining the specific contribution of child and parent factors to differences between families in their adjustment to DL. Understanding which families have experienced the most problems is essential to prevent long-term effects of the COVID-19 pandemic on both children and their parents, and to be well-prepared for possible future school lockdowns.

During the COVID-19 pandemic, the governments of 188 countries imposed school lockdowns which severely modified the education for over 1.7 billion children and adolescents worldwide. This decision, although perhaps necessary, disrupted the daily lives of many families because school is an essential source of physical and mental health; indeed, the general lockdown had a profound and complex negative impact on families (2). For children, school closures had a negative effect not only on learning (4), but also on psychological health (2). More specifically, previous research (3) found that between 17.4 and 27.6% of parents reported general negative experiences related to DL for their child. In addition, high rates of symptoms of depression (22.6–43.7%) and anxiety (18.9–37.4%) among children and adolescents have been reported during the pandemic (2). For parents, school closures were highly challenging due to the need to take on the role of being both a teacher and parent for the child. Calvano et al. (5) investigated a range of different stressors for German parents during the COVID-19 pandemic, and found that school closures were one of the most challenging, with as many as 56% reporting high or extremely high burden. In addition, Thorell et al. (3) showed that a substantial proportion of parents reported negative experiences linked to DL, with worrying and stress exceeding 40% of families across several European countries. Interestingly, some studies also reported positive experiences related to DL for both children and their parents (3, 6). Due to the COVID-19 pandemic, several external stressors for parents (e.g., business meetings, guests, business trips) have disappeared. In addition, mastering the challenges of the COVID-19 situation together may have strengthened family cohesion (6). Finally, children troubled by school due to bullying or other stressors may have experienced the situation of DL as relieving (6). Overall, these findings emphasize that families varied greatly with regard to their experiences of DL, but little is known about specific child and parental factors contributing to these differences.

Previous studies have underlined that background variables such as socio-economic status (SES) and child age and sex are of importance in managing critical situations (7). As for child factors, executive functions (EF) has been found to be strongly related to general psychosocial adjustment (8, 9) and academic achievement [e.g., (7–9)]. With regard to parental factors, psychological well-being, specifically positive mental health, has been shown to play a crucial role in people's positive adjustment during times of crisis (10).

The overall aim of the present study was therefore to examine whether background factors (i.e., child age, child sex, and SES), children's EF deficits and parents' psychological well-being were associated with negative and positive effects of DL during the COVID-19 pandemic.

### Background Factors and DL-Related Outcomes

Albeit important, many previous studies examining the psychological effects of the COVID-19 pandemic did not examine the role of background factors (2), such as child age, child sex and family's SES (7). SES has commonly been indexed by family income or parental educational level. However, previous research has shown that parental educational level is more strongly associated with children's academic outcomes compared to income (11). A few studies have shown larger DL-related negative effects for younger compared to older children (3), and larger lockdown-related effects on families with low compared to high parental educational level (12). With regard to child sex, whilst in developing countries there are larger negative effects of DL on girls because of a disproportionate increase in unpaid household work (13), in Western countries we can expect smaller or no differences between boys and girls.

### Executive Function Deficits and DL-Related Outcomes

EF is an umbrella term for higher-order cognitive functions required to direct behavior toward a goal (14). It includes inhibition (i.e., the ability to inhibit dominant responses), working memory (i.e., maintaining task-relevant information in mind), shifting (i.e., switching between different tasks), and planning (i.e., choosing the necessary actions and the right order to reach a goal) (14). The ability to direct behavior toward a goal is a key to successfully complete most academic tasks (15), and executive functioning is therefore strongly related to academic achievement (16–18). During school closures, the demands on executive skills have most likely increased as DL requires the child to plan his/her own schoolwork to a much greater extent compared to normal schooling, to maintain attention even though the teacher is only shown on the screen or not at all, and to inhibit the home-environmental distractions during online lessons. In line with this, Hai et al. (19) found significant associations between EF deficits and difficulties adjusting to DL. However, these results are limited to children with Attention Deficit Hyperactivity Disorder (ADHD).

### Psychological Well-Being and DL-Related Outcomes

Positive mental health, a key aspect of psychological well-being, can be defined as the presence of general emotional, psychological, and social well-being (20). Individuals characterized by positive mental health typically have a high sense of control and can adaptively cope with unexpected situations (21). Previous research also found associations between high parental self-efficacy and greater family well-being due to the preserved ability to provide competent, high-quality parenting even when faced with challenges and adversity (10). Good parenting skills become particularly crucial when children are confined at home. In line with this, less parental coping skills in relation to lockdown measures were associated with higher parental stress, poorer parent-child relations, and increased child behavioral problems (5). In an Italian sample involving parents of first grade children, the association between parents' difficulties with managing their children's DL and perceived stress was no longer significant when taking the effect of parental self-efficacy into account (22). In addition, positive mental health assessed before the pandemic has been identified as a predictor of lower burden during the first phase of the COVID-19 pandemic (23). However, the role of positive mental health for parents in relation to DL and the independent effects on parent and child factors on DL during COVID-related school lockdown has not yet been investigated.

### Aim of the Present Study

As described above, previous research has demonstrated that DL during the COVID-19 pandemic has had an important impact on both children (1–3) and their parents (3, 5, 22). However, there is a lack of knowledge concerning the specific factors that could explain differences between families. Understanding the sources of variability in outcomes related to DL in times of school closure is crucial to provide support based on the needs of individual families. Thus, the aim of the present study was to examine the contribution of background factors (i.e., child age, child sex, and parental educational level), child factors (i.e., children's executive deficits), and parent factors (i.e., parents' psychological well-being) to both positive and negative effects of DL during the COVID-19 pandemic.

Based on previous research (3), we expected lower child age to be related to more negative effects of DL on both children and their parents. Considering the few, inconsistent results of previous research examining the effects of child sex on DL, we did not formulate any a priori hypothesis in this regard. In relation to parental educational level, we expected lower parental education to be linked to more negative effects of DL for both parents and their children (12, 24). In terms of child factors, we expected that EF deficits would be associated with DL-related negative outcomes for the child (19). In terms of parent factors, parents' psychological well-being would primarily be related to less DL-related negative outcomes on parents (23) and to positive effects of DL on family (10).

## Methods

### Participants and Procedure

All participants of the present study were part of an international study (3) conducted in seven European countries with the aim to investigate parental experiences of DL due to school closures during the early phase of the COVID-19 pandemic (i.e., April through June, 2020). Inclusion criteria for the present study were: (1) being the parent of a child (aged 6–19 years) receiving DL due to school closure during the COVID-19 pandemic; (2) being Italian (i.e., living in Italy and speaking mostly or only Italian in the home setting); (3) having a child without any mental health problems. If parents had more than one child receiving DL, they were asked to respond to the survey referring to their oldest child.

For the purpose of this study, we focused on parents of children with typical development (*n* = 667), excluding reports from parents who had a child with mental health problems. In addition, to ensure the accuracy of data analysis, questionnaires with more than 25% of missing data were considered invalid and not included. This resulted in a few participants (30/667) being excluded. The final sample included 637 participants. More specifically, we included 587 (92%) mothers and 45 (7%) fathers (5 respondents chose to not report their gender). Parental educational level (based on both parents) was up to 8th grade for 66 (11%) families, up to high-school diploma for 276 (43%) families, up to a bachelor's degree for 236 (37%) families, and up to a master's degree or a Ph.D. for 59 (9%). Target children were 304 (48%) males and 329 (52%) females (4 families did not report their child's gender) aged between 6 and 19 years (*M* = 10.8, *SD* = 3.24), with 339 (53%) children attending 1st to 5th grade, 183 (29%) attending 6–8th grade, and 115 (18%) attending high school.

Parents reported that children (1st−8th grade) spent on average 4 h/day on DL, whereas adolescents (9–13th grade) spent on average 5 h/day on DL. For children, 35% of the time devoted to schoolwork was spent on self-studies, 35% in contact with a parent, 24% in contact with a teacher, and 7% in contact with peers. Adolescents spent 45% of the time devoted to schoolwork on self-studies, 40% in contact with a teacher, 8% in contact with peers, and 7% in contact with a parent.

Data were collected using an anonymous digital survey distributed *via* social media, schools, and parent networks. Several schools from different socio-economic areas were asked to support the study by distributing the link. The study was approved by the ethics committee of the School of Psychology at University of Padua (protocol no. 3620). Written informed consent was obtained from parents before they took part in the study.

### Materials

The online questionnaire was originally created for the cross-cultural study (3) and focused on several aspects of parents' experiences of DL. In the present study, we included three domains: negative effects of DL on children, negative effects of DL on parents, and positive effects of DL on the family. Items were developed based on a previous qualitative study (unpublished data) which examined what aspects of family functioning that parents thought were most strongly affected by school closure during the COVID-19 pandemic. In addition, the present study included a measure of children's EF deficits and a measure of parents' psychological well-being. These measures are described in more detail below.

#### Negative Effects of DL on Children

The following five items measured parents' perceptions of negative effects of DL for the child: (1) “My child finds particularly difficult to sustain his/her attention when schooling takes place from home”; (2) “During homeschooling, my child often gets distracted by other things when s/he/ should be studying”; (3) “Homeschooling puts too high demands on the child to plan his/her own schoolwork”; (4) “For my child is impossible to work well because of homeschooling”; (5) “Homeschooling has negative effects on the child's life”. Each item was rated on a scale ranging from 1 (“strongly disagree”) to 5 (“strongly agree”), with higher scores indicating more negative effects for the child. Cronbach's alpha was 0.82.

#### Negative Effects of DL on Parents

The following five items measured parents' perceptions of negative effects of DL for themselves: (1) “As a parent, I need to take an active part in homeschooling to make sure that my child is doing the work that s/he is supposed to do”; (2) “My child has difficulties with carrying out homeschooling without having an adult at home who can support him/her”; (3) “I feel stressed because of the extra work that homeschooling demands of me as a parent”; (4) “I am worried that my child will not be able to handle school as well as s/he normally does because of homeschooling”; (5) “Homeschooling has had negative effects on my own life”. Each item was rated on a scale ranging from 1 (“strongly disagree”) to 5 (“strongly agree”), with higher scores indicating more negative effect of DL for parents. Cronbach's alpha was 0.88.

#### Positive Effects of DL on Family

The following three items measured parents' perceptions of positive experiences of DL: (1) “I see certain advantages with the fact that my child is homeschooled”; (2) “Homeschooling has positive effects on the child's life”; (3) “Homeschooling has positive effects on my own life”. Each item was rated on a scale ranging from 1 (“strongly disagree”) to 5 (“strongly agree”), with higher scores indicating greater positive effects. Cronbach's alpha was 0.79.

#### Background Variables

Parents were asked to report their child's age and sex. Moreover, parental educational level for both the child's mother and father was measured using a 4-point scale (1 = completed <8th grade; 2 = completed some years of high school, without obtaining the high-school diploma; 3 = high-school diploma and/or some years of university; 4 = master's degree or a Ph.D.). In our analyses, we averaged the score for the mother and the father.

#### Children's Executive Function Deficits

Children's EF deficits were measured using an abbreviated (8 items) version of the Childhood Executive Functioning Inventory (CHEXI; 25). The CHEXI is freely available in many different languages (www.chexi.se), and previous studies have shown that this questionnaire has good test-retest reliability (25). It has also been shown to be related to daily life functioning (e.g., academic achievement) (26). The CHEXI includes two subscales measuring working memory (e.g., “My child has difficulty remembering lengthy instructions”) and inhibition (e.g., “My child has difficulty stopping an activity immediately upon being told to do so”). Each item is rated on a scale ranging from 1 (“definitely not true”) to 4 (“definitely true”), with higher scores indicating greater EF deficits. Parents were asked to report their child's executive functioning during the last 6 months. In this study, we used the short version to keep the number of items as low as possible and thereby hopefully optimize the response rate. Cronbach's alpha was 0.90.

#### Parents' Psychological Well-Being

The Positive Mental Health scale (PMH-scale; (20) was used to assess psychological well-being and positive mental health in parents. The scale assessed emotional, psychological and social aspects of individual well-being. Participants rated statements such as “I enjoy my life”, “In general I am confident”, “I am in a good physical and emotional condition” on a 4-point Likert scale ranging from 1 (“do not agree”) to 4 (“agree”). Higher scores indicated more positive mental health. Cronbach's alpha was 0.81.

### Statistical Analyses

Data analyses were conducted using R (27). First, all variables included in the study were standardized. Second, Pearson correlations were used to investigate intercorrelations among all variables. Third, three hierarchical linear regression analyses were run to evaluate the specific contribution of the independent variables in relation to DL-related negative effects (on either children or parents) and DL-related positive effects. The order of predictors was selected *a priori*. In the first step, we entered child sex and age to test for their effects on DL-related effects and to control for their association with the other predictors (i.e., EF deficits and parents' psychological well-being). Children's EF deficits were then entered in the second step, as previous research has emphasized that executive functioning is fundamental for academic achievement (18). Finally, parents' psychological well-being was entered in the last step, as parental functioning was likely to play an important role in the adjustment to lockdown measures, specifically with regard to DL previous research has shown that parents had to take on responsibility for schooling due to the lack of online teaching (3, 5, 10). Finally, the interaction effect of EF deficits and psychological well-being was also examined in relation to all three outcomes.

## Results

Table 1 presents correlations among all variables (DL-related effects, child age and sex, parental educational level, children's EF deficits and parents' psychological well-being). The results showed that negative effects of DL on parents and children were strongly associated, and both these variables were also negatively associated with positive effects of DL. Child age was significantly associated with all other variables, with younger age being related to more negative effects on both parents and children and less positive effects. Child sex was significantly associated with negative effects of DL and EF deficits, male being related to more negative effects and higher EF deficits. Parental educational level was negatively associated with child age and positively associated with parents' psychological well-being, but it was not significantly associated with any DL-related effects. Hence, parental educational level was not included in the regression models. With regard to associations between child/parent factors and DL-related effects, all three outcome variables were significantly associated with both children's EF deficits and parents' psychological well-being.

**Table 1:** Descriptive statistics and correlations among study variables (*N* = 637).

	**2**	**3**	**4**	**5^#^**	**6**	**7**	**8**	**Mean (SD)**
Negative effects of DL on child	0.76^***^	−0.48^***^	−0.28^***^	0.08^*^	−0.07	0.55^***^	−0.19^***^	2.90 (0.94)
Negative effects of DL on parents		−0.48^***^	−0.50^***^	0.11^**^	0.05	0.53^***^	−0.24^***^	3.37 (1.12)
Positive Effects of DL			0.28^***^	−0.02	−0.02	−0.22^***^	0.22^***^	2.19 (0.86)
Child age				−0.09^*^	−0.16^***^	−0.33^***^	0.09^*^	10.80 (3.24)
Child sex^#^					0.07	0.16^***^	−0.06	–
Parental educational level						−0.04	0.08^*^	2.63 (0.76)
Children's EF deficits							−0.33^***^	19.10 (6.78)
Parents' psychological well-being								26.07 (4.41)

* *p < 0.05*,

** *p < 0.01*,

*** *p < 0.001; DL, distance learning; EF, executive functions*.

# *Point-biserial correlation*.

Next, we conducted a hierarchical regression analysis for each dependent variable^1^: negative effects of DL on child, negative effects of DL on parent, and positive effects of DL on family (see Table 2). With regard to negative effects of DL on children, the background variables included in the first step explained 8% of the variance. In the second step, the effect of EF deficits was significant, with this step explaining 22% of the variance. Parents' psychological well-being (entered in the third step) had no significant effect. With regard to negative effects of DL on parents, the background variables explained 25% of the variance. EF deficits had a significant effect, explaining 14% of the variance. Parents' psychological well-being also had a significant effect, but it explained only an additional 2% of the variance. Finally, for positive effects of DL, background variables explained 7% of the variance. In the second step, the effect of EF deficits was significant and explained 2% of the variance. In the third step, parents' psychological well-being was significant but only explained 3% of the variance. Thus, in total, the variables included in the present study could only explain 12% of the variance in positive effects of DL. No significant interaction effects of children's EF deficits and parents' psychological well-being were found for any of the three dependent variables.

**Table 2:** Hierarchical multiple regression analysis with negative effects (on children and parents) and positive effects of DL as the dependent variables and background factors, EF deficits and parent's psychological well-being as independent variables (*N* = 637).

	**Negative effects of DL on children**			**Negative effects of DL on parent**			**Positive effects of DL**		
	**β**	**Adjusted*R*^2^**	**Δ*R*^2^**	**β**	**Adjusted*R*^2^**	**Δ*R*^2^**	**β**	**Adjusted*R*^2^**	**Δ*R*^2^**
**Step 1**		0.08			0.25			0.07	
Age	−0.28^***^			−0.50^***^			0.28^***^		
Child sex	0.06			0.07			0.01		
**Step 2**		0.30	0.22		0.39	0.14		0.09	0.02
Children's EF deficits	0.51^***^			0.38^***^			−0.08^*^		
**Step 3**		0.31	0.01		0.41	0.02		0.12	0.03
Parents' psychological well-being	−0.01			−0.08^*^			0.17^***^		

* *p < 0.05*,

*** *p < 0.001; EF, executive functions*.

## Discussion

Several recent investigations have explored the negative impact of DL on psychological health, but few studies have examined the extent to which child and parent factors contribute to differences between families regarding their experiences of DL. Understanding the specific child and parent factors that impact on parents' management of their children's DL is essential to tailor interventions aimed at reducing possible long-term effects related to DL. The main goal of the present study was to investigate the role of background factors, child factors, and parental factors in explaining differences in the perception of negative and positive effects of DL due to school closures imposed during the first wave of the COVID-19 pandemic. Specifically, three outcomes were assessed: negative effects of DL on children, negative effects of DL on parents, and positive effects of DL on the family. Regarding DL-related negative effects, we found that both background factors and children's EF deficits explained most part of the variance, but there were some noteworthy differences. For DL-related negative effects on children, EF deficits were the most important factor, whereas child age was the most important factor for negative effects on parents. Regarding DL-related positive effects, all variables explained only a small part of the variance. Child age was the most important factor, but children's EF deficits and parents' psychological well-being also had a significant impact.

### The Role of Background Factors

The present study examined several background variables (i.e., child age and sex, and parental educational level) and how they contributed to Italian families' adjustment to DL. There is ample evidence suggesting that the effects of stressful events vary significantly depending on the age of the child and the family's SES. More specifically, younger children are more likely to be affected by their parents' stress generated by the pandemic, and parental stress has been found to be linked to more child behavior problems at school (29). Moreover, children and adolescents with low family SES seem to have more difficulties coping with stressful life situations than their peers with high SES (7). In the present study, we assessed SES as parental educational level, since previous research has found parental education to be most strongly associated with children's academic outcomes (11). With regard to effects of age, our results are consistent with at least one previous study finding larger negative effects for families with younger compared to older children (3). Child age had a particularly strong impact on DL-related negative effects on parents. Generally, during the lockdown, parents had to meet various demands simultaneously: homeworking, financial difficulties, and loss of social support (5). Moreover, parents of younger children had to take over the role of teachers due to children's difficulties with self-regulation, attentional focusing, academic motivation, and limited autonomy with managing electronic devices involved during DL-related activities. Thus, younger children required greater support from their parents to cope well with DL (30).

Surprisingly, parental educational level was not significantly associated with DL-related effects within the present study, and this variable was therefore not further considered in the regression analyses. This finding is inconsistent with previous studies showing that families with lower (vs. higher) parental educational level reported a stronger impact on DL-related effects (12). A possible explanation may be the homogeneity in parental education in our sample, with families with a low parental education being underrepresented.

Finally, parents of boys experienced greater negative effects compared to parents of girls. This was expected, considering that previous research has shown that girls performed significantly better than boys during DL (31, 32), probably due to a higher motivation for learning and more functional study habits. However, it should be noted that the effect of child sex was negligible in our study.

### The Role of Children's EF Deficits

Our study showed that children's EF deficits were strongly associated with DL-related negative effects on both children and parents. This finding is in line with previous research showing that children's EF deficits have an adverse effect on academic achievement [e.g., (7–9)], and that children with preexisting EF deficits are more vulnerable to the negative psychological impact of the COVID-19 pandemic (33). In addition, this pattern is consistent with studies showing that children with neurodevelopmental disorders known to be related to EF deficits (e.g., ADHD) found DL particularly challenging [e.g., (20, 34)]. Our findings highlight that variation in EFs in non-clinical samples is also of relevance. Thus, following the Research Domain Criteria [RDoC; (35)], efforts should be directed toward the identification of underlying deficits (e.g., EF deficits) in order to provide individualized support, rather than assuming that all children with or without a certain disorder have the same difficulties. The results of the present study also showed that children's EF deficits are an important factor contributing to DL-related negative outcomes on parents, possibly due to the higher demands on children's executive skills in the context of DL activities such as self-study and the ability to plan one's own schoolwork (3). Thus, children with more EF deficits may require more assistance from their parents to perform adequately (36), therefore increasing parents' perception of negative effects on their life related to DL.

### The Role of Parents' Psychological Well-Being

Previous research has shown that parents' psychological well-being, self-efficacy, and positive mental health are essential aspects when facing challenges and adversity (10, 22, 23). Individuals with better positive mental health before the pandemic reported lower burden during the first phase of the COVID-19 outbreak (23). However, this last study did not investigate effects related to DL, nor the independent effects of parents' psychological well-being when controlling for other factors. In the present study, we found a significant negative association between parents' psychological well-being and DL-related negative effects on both children and their parents, but this association did not remain significant for DL-related effects on children when including EF deficits in the previous step of the regression analysis. This shows that there is an overlap between EF deficits and parents' psychological well-being, especially in relation to effects on children. As regards DL-related positive effects, our research underlined the major role of parents' positive mental health. Altogether, our findings are consistent with previous studies (5, 10, 23) suggesting that greater parental self-efficacy and better parental coping with lockdown measures are related to more positive parental experiences of DL and increased family well-being. Moreover, our study expands the results of previous work on Italian parents (22), which found that the association between parents' difficulties in managing their children's DL and levels of perceived stress was no longer significant after controlling for parental self-efficacy.

### Strengths and Limitations

The present study had several strengths, the inclusion of large sample of Italian families with at least one child experiencing DL during the first wave of the COVID-19 pandemic; the collection of data during school closures due to the national lockdown imposed by the Italian government, rather than the reliance on retrospective reports; and the inclusion of three different measures of DL-related effects on families—negative impact on children, negative impact on parents, as well as positive impact. Albeit sharing some variance, the DL-related effects were specific as suggested by the fact that different factors (i.e., background variables, child factors, and parental factors) contributed in unique ways to the three outcomes (37). With regard to limitations, we relied on cross-sectional data rather than investigating abilities before and during DL and the direction of the effects could therefore not be established. For instance, it is possible that DL-related negative effects cause lower levels of parental psychological well-being, rather than the other way around. In support of this view, previous research [e.g., (37)] has shown that DL during the COVID-19 pandemic has led to increased parental stress, which in turn decreased parents' psychological well-being. Reciprocal relations indicating that parents' psychological well-being and their effects of DL influence each other over time are also possible. Secondly, we relied on parents' perceptions of child outcomes rather than on children's own reports. This was necessary as we included children as young as 6 years of age. In addition, self-reports could be problematic as children may struggle to describe their difficulties *via* a self-report measure, choosing extreme options and basing their responses on a single experience (38). Adults more often judge their experiences holistically, and parental reports should therefore be regarded as a primary source of information about children's adjustment. Thirdly, we did not use validated measures to assess DL-related effects. However, we developed the items based on the results from a small qualitative study in which parents were asked to describe the effects of DL on family functioning. Based on the results of this study, it became clear that the COVID-19 pandemic is a unique event that has posed new challenges, and available questionnaires were therefore not able to evaluate the most relevant aspects. More research is warranted to evaluate the psychometric properties of the measures included in the present study. Finally, despite attempts to recruit participants *via* schools in a range of different socio-economic areas, families with a low level of parental education were underrepresented.

### Conclusions and Future Directions

The present study offers a comprehensive investigation of the contribution of background factors, children's EF skills, and parents' psychological well-being in relation to DL-related effects. After the inclusion of background variables, often omitted in prior work, we found more severe impacts on families with a younger compared to an older child. For negative DL-related effects on parents, the age of the child was the most important variable. Children's EF deficits were the variable that was most strongly related to negative DL-related effects on children, but played a key role also for negative effects on parents. Moreover, high levels of parental psychological well-being seemed to work as a protective factor. As a next step, studies might assess long-term effects and consider other factors that could potentially influence the experience of DL, such as parenting style, parents' availability in supporting their children's DL, quality of sleep, school schedules, and previous academic performance. Our finding that EF deficits significantly contributed to DL-related negative effects on children suggests that future research might consider how to best limit the negative consequences of EF deficits in the school setting in case of future school closures. For instance, previous studies conducted before the pandemic have shown that reducing task length, dividing tasks into sub-units, giving explicit instructions, providing help with organizing school work, and regular feedback from an adult or peer are effective strategies (26, 36). Through a more complete understanding of the complex relation between background factors and child and parent factors, we are better equipped to provide individualized support to families and thereby hopefully reduce long-term negative effects of school closures during the COVID-19 pandemic on children's learning and family life.

## Data Availability Statement

The raw data supporting the conclusions of this article will be made available by the authors, without undue reservation.

## Ethics Statement

The studies involving human participants were reviewed and approved by University of Padua, Padua, Italy. The patients/participants provided their written informed consent to participate in this study.

## Author Contributions

LT developed the study concept. GC performed the testing. GC, IM, and LT performed the data analysis. GC and IM drafted the manuscript. UM, MR, and LT provided revisions. All authors contributed to the article and approved the submitted version.

## Conflict of Interest

The authors declare that the research was conducted in the absence of any commercial or financial relationships that could be construed as a potential conflict of interest.

## Publisher's Note

All claims expressed in this article are solely those of the authors and do not necessarily represent those of their affiliated organizations, or those of the publisher, the editors and the reviewers. Any product that may be evaluated in this article, or claim that may be made by its manufacturer, is not guaranteed or endorsed by the publisher.

## Footnotes

^1^We evaluated if the assumptions of our regression model were met, using a procedure recommended by Peña & Slate (28) *via* the gvlma package. We evaluated assumptions of linearity, homoscedasticity, uncorrelatedness, and normality on the residuals of our final regression model. Regarding negative effects of DL on children the Global test indicated that the assumptions of the regression model could not be rejected, χ^2^(4) = 6.93; *p* = 0.29 (skewness: χ^2^(4) = 0.25, *p* = 0.97; curtosis: χ^2^(4) = 0.34, *p* = 0.90; link function: χ^2^(4) = 0.14, *p* = 0.06; homoscedasticity: χ^2^(4) = 0.19, *p* = 0.61). Regarding negative effects of DL on parent the Global test indicated that the assumptions of the regression model could not be rejected, χ^2^(4) = 5.06; *p* = 0.28 (skewness: χ^2^(4) = 0.002, *p* = 0.96; curtosis: χ^2^(4) = 0.001, *p* = 0.98; link function: χ^2^(4) = 1.49, *p* = 0.22; homoscedasticity: χ^2^(4) = 3.56, *p* = 0.06). Regarding positive effects of DL the Global test indicated that the assumptions of the regression model could not be rejected, χ^2^(4) = 7.22; *p* = 0.13 (skewness: χ^2^(4) = 0.37, *p* = 0.23; curtosis: χ^2^(4) = 0.87, *p* = 0.09; link function: χ^2^(4) = 0.03, *p* = 0.08; homoscedasticity: χ^2^(4) = 0.68, *p* = 0.09). Thus, our model met the assumptions required to perform regression analysis.
